# Modulation of Redox and Inflammatory Signaling in Human Skin Cells Using Phytocannabinoids Applied after UVA Irradiation: In Vitro Studies

**DOI:** 10.3390/cells13110965

**Published:** 2024-06-03

**Authors:** Adam Wroński, Iwona Jarocka-Karpowicz, Arkadiusz Surażyński, Agnieszka Gęgotek, Neven Zarkovic, Elżbieta Skrzydlewska

**Affiliations:** 1 Dermatological Specialized Center “DERMAL” NZOZ in Białystok, Nowy Swiat 17/5, 15-453 Bialystok, Poland; adam.wronski@dermal.pl; 2 Department of Analytical Chemistry, Medical University of Bialystok, A. Mickiewicza 2D, 15-222 Bialystok, Poland; iwona.jarocka-karpowicz@umb.edu.pl (I.J.-K.); agnieszka.gegotek@umb.edu.pl (A.G.); 3 Department of Medicinal Chemistry, Medical University of Bialystok, Kilinskiego 1, 15-069 Bialystok, Poland; arkadiusz.surazynski@umb.edu.pl; 4 Laboratory for Oxidative Stress, Rudjer Boskovic Institute, Bijenicka 54, HR-10000 Zagreb, Croatia; zarkovic@irb.hr

**Keywords:** fibroblasts, inflammation, keratinocytes, phytocannabinoids, redox balance, UVA radiation

## Abstract

UVA exposure disturbs the metabolism of skin cells, often inducing oxidative stress and inflammation. Therefore, there is a need for bioactive compounds that limit such consequences without causing undesirable side effects. The aim of this study was to analyse in vitro the effects of the phytocannabinoids cannabigerol (CBG) and cannabidiol (CBD), which differ in terms of biological effects. Furthermore, the combined use of both compounds (CBG+CBD) has been analysed in order to increase their effectiveness in human skin fibroblasts and keratinocytes protection against UVA-induced alternation. The results obtained indicate that the effects of CBG and CBD on the redox balance might indeed be enhanced when both phytocannabinoids are applied concurrently. Those effects include a reduction in NOX activity, ROS levels, and a modification of thioredoxin-dependent antioxidant systems. The reduction in the UVA-induced lipid peroxidation and protein modification has been confirmed through lower levels of 4-HNE-protein adducts and protein carbonyl groups as well as through the recovery of collagen expression. Modification of antioxidant signalling (Nrf2/HO-1) through the administration of CBG+CBD has been proven to be associated with reduced proinflammatory signalling (NFκB/TNFα). Differential metabolic responses of keratinocytes and fibroblasts to the effects of the UVA and phytocannabinoids have indicated possible beneficial protective and regenerative effects of the phytocannabinoids, suggesting their possible application for the purpose of limiting the harmful impact of the UVA on skin cells.

## 1. Introduction

Several studies conducted in recent years indicate that synthetic medicinal remedies used in the pharmacotherapy of various skin diseases are increasingly replaced by preparations based on compounds/substances of natural origin, mainly plants, that are better absorbed and have fewer side effects [1]. This applies primarily to compounds with antifungal, anti-inflammatory, and antioxidant properties [2]. Popular plants are increasingly being taken into account in the pharmacotherapy of skin problems, including cannabis, especially *Cannabis sativa* L., which contains many biologically active compounds, especially a significant group of compounds that do not have psychoactive effects but have protective/regenerative properties for skin cells [3]. This particularly applies to the phytocannabinoids that are characterised by a high structural and functional similarity with endogenous lipid mediators—endocannabinoids—and may, therefore, modulate various metabolic pathways in human cells [4], inter alia, by means of activating G-protein-coupled receptors [5]. This is mainly the case with the activation of cannabinoid receptors (CB1/2) that are involved in the regulation of inflammation by modifying the level of TNFα and redox balance as well as the level of ROS [6]. The levels of the above parameters also change as a result of cell exposure to UV radiation [7].

Both phytocannabinoids and their 2-3-component systems were tested for their potential therapeutic use in lymphoma, glioblastoma multiform, and leukaemia [8,9,10]. The group of the best known and therapeutically used phytocannabinoids includes cannabidiol (CBD), which has antioxidant, anti-inflammatory, anticancer, and antibacterial properties [11,12,13]. Another representative of phytocannabinoids, a precursor of other compounds in this group, is cannabigerol (CBG) [14], which may modulate cell metabolism through mechanisms that have not been precisely clarified. The biological effects of CBD and CBG result from their structure, notably, the presence of hydroxyl groups attached to the aromatic ring (easily oxidised to the quinone form), double bonds, and the pentyl chain [15,16]. 

One of the physical factors that modifies the metabolism of skin cells on a daily basis is the power of sunlight—ultraviolet (UV)—radiation [17]. More than 90% of solar UV radiation consists of UVA (315–400 nm) that can penetrate the epidermis and dermis, causing changes in the metabolism of skin cells in those layers [18]. The energy of the UVA may directly oxidise cellular molecules or may be absorbed by cellular chromophores that further interact with molecular oxygen and produce reactive oxygen species (ROS), including hydrogen peroxide, superoxide, and hydroxyl radicals [19]. Consequential changes in the structure and functions of cellular antioxidants [20] promote metabolic disorders both at the level of keratinocytes and skin fibroblasts [21]. It has been shown that both CBG and CBD may regulate the redox balance by inhibiting the generation of ROS and reactive nitrogen species (RNS) in the skin fibroblasts [22]. Moreover, by reducing the effectiveness of the nuclear factor NFκB (nuclear factor kappa-light-chain-enhancer of activated B cells) that is responsible for, among other things, the biosynthesis of proinflammatory cytokines TNFα (tumour necrosis factor α) and IL-1β (interleukin-1β), those phytocannabinoids could reduce inflammation in the UV-irradiated human skin, the CBD-affecting keratinocytes, and fibroblasts, whereas the only affected keratinocytes [14,23,24]. Moreover, CBG, when applied to human skin, reduces trans-epidermal water loss better than CBD, which may improve the skin barrier [22]. However, there are no data about the effects of CBG, used alone or in combination with other phytocannabinoids, on the metabolism of skin cells under the conditions of oxidative stress caused by physicochemical factors, including UV radiation. Since the physicochemical features of CBG resemble those of CBD, while concentrations of the biologically effective CBG and CBD differ [25], the combined use of both phytocannabinoids might be more effective for skin cells than CBG or CBD given alone. 

Therefore, the aim of this study was to compare the effects of CBD or CBG on the redox balance and proinflammatory signalling of in vitro cultured human keratinocytes and fibroblasts exposed to the UVA radiation, and, for the first time, to determine the areas of synergism in which those compounds may act in the context of the examined skin cells.

## 2. Materials and Methods

### 2.1. Materials

#### Cells Cultures

The following human skin cells obtained from the American Type Culture Collection (ATCC, Manassas, VA, USA) were used in this study: human keratinocytes (CDD 1102 KERTr – CRL-2310) and fibroblasts (CCD-25Sk - CRL-1474).

### 2.2. Methods

#### 2.2.1. Cells Cultures

The keratinocytes were cultured in serum-free medium (SFM) with 1% Bovine Pituitary Extract and human recombinant Epidermal Growth Factor. The fibroblasts were cultured in Dulbecco’s Modified Eagle Medium containing 10% foetal bovine serum (FBS) and antibiotics (penicillin (50 U/mL) and streptomycin (50 μg/mL) under sterile conditions (ambient air with 5% CO_2_, temperature of 37 °C). Sterile cell culture reagents were obtained from Gibco (Grand Island, NY, USA). 

#### 2.2.2. Cells Treatment

After reaching 70% confluence, the cells were washed with phosphate-buffered saline (PBS) and suspended in this buffer at 4 °C. The total radiation (UVA) dose was 30 J/cm^2^ for keratinocytes and 20 J/cm^2^ for fibroblasts using Bio-Link Crosslinker BLX 312/365 (Vilber Lourmat, Eberhardzell, Germany). Cells were irradiated on ice to eliminate heat stress from irradiation. The exposure doses were chosen to correspond to 75 ± 5% cell (keratinocytes and fibroblasts) viability as measured using the MTT (3-(4,5-dimethylthiazolyl-2)- 2,5-diphenyltetrazolium bromide) test [26]. The control cells were incubated in parallel without irradiation. In order to assess the effect of the phytocannabinoids on cellular metabolism, keratinocytes and fibroblasts were treated for 24 h with cannabigerol (CBG-1µM; Cayman Chemical Company, Ann Arbor, MI, USA), cannabidiol (CBD-5µM; THC Pharm GmbH, Frankfurt, Germany), or both phytocannabinoids (CBG-1µM+CBD-5µM). A detailed description of solutions prepared for the experiment purposes was presented in the work by Wroński et al. [25]. In short, the CBG and CBD stock solution (32 mM each) was prepared in ethanol (99.8%) in order to subsequently dilute that solution with ethanol to a concentration of 0.33 mM (CBG) and 1.6 mM (CBD); then, it was added to the culture medium to obtain a concentration of 1 µM and 5 µM for the CBG and CBD, respectively. Due to both phytocannabinoids’ (CBG and CBD) solubility, all media contained ethanol to maintain the same conditions for all experimental cells (with the final ethanol concentration in the medium at 0.3%). 

In order to assess the effect of CBG and CBD on the cellular metabolism of keratinocytes and fibroblasts unexposed and exposed to the UVA, the cells were divided into two skin cell groups and treated as follows:

- Control—*keratinocytes/fibroblasts* incubated for 24 h in medium only under standard conditions;
- CBG—*keratinocytes/fibroblasts* incubated for 24 h in medium with CBG (1 µM);
- CBD—*keratinocytes/fibroblasts* incubated for 24 h in medium with CBD (5 µM);
- CBG+CBD—*keratinocytes/fibroblasts* incubated for 24 h in medium with CBG-1 µM+CBD-5 µM;
- UVA—*keratinocytes* exposed to UVA(30 J/cm^2^)/*fibroblasts* exposed to UVA(20 J/cm^2^);
- UVA+CBG—*keratinocytes* exposed to UVA(30 J/cm^2^)/*fibroblasts* exposed to UVA(20 J/cm^2^) and, after that, all cells were incubated for 24 h in medium with CBG(1 µM);
- UVA+CBD—*keratinocytes* exposed to UVA(30 J/cm^2^)/*fibroblasts* exposed to UVA(20 J/cm^2^) and, after that, all cells were incubated for 24 h in medium with CBD(5 µM);
- UVA+CBG+CBD—*keratinocytes* exposed to UVA(30 J/cm^2^)/*fibroblasts* exposed to UVA(20 J/cm^2^) and, after that, all cells were incubated for 24 h in a medium with CBG-1 µM+CBD-5 µM.

After incubation, the cells were rinsed with cold PBS buffer (4 °C), scraped from the dishes, and then sonicated on ice and centrifuged for 15 min at 12,000× *g* at 4 °C (to separate the membrane fraction). Supernatants were collected for analysis purposes, and the results obtained were normalised to the total protein content measured by means of the Bradford test [27].

#### 2.2.3. Prooxidative Parameters

In order to determine the activity of NADPH oxidase (NOX-EC 1.6.3.1), the luminescence method was used as previously described [28] and was expressed in terms of relative luminescence units (RLU) per milligram of protein.

Determination of total ROS generation was performed in keratinocytes and fibroblasts using an electron spin resonance (ESR) spectrometer (Noxygen GmbH/Bruker Biospin GmbH, Rheinstetten, Germany) based on the method recommended by Misra and Fridovich [29] and was expressed in terms of micromoles per minute per milligram of protein.

#### 2.2.4. Antioxidant Enzymes Activity

Cytosolic superoxide dismutase activity—SOD-1 (Cu,Zn-SOD;C.1.15.1.1) [30] and manganese-dependent superoxide dismutase—SOD-2 (Mn-SOD;EC.1.15.1.1) [31] were determined by means of the spectrophotometric method at a wavelength of 480 nm as presented in the previous work. One unit of SOD was defined in terms of the amount of enzyme that inhibited the oxidation of epinephrine to adrenochrome by 50%. SOD-1 and SOD-2 activities were determined in terms of U per milligram of protein. 

Catalase activity (CAT–EC.1.11.1.9) was determined spectrophotometrically by measuring the decrease in absorbance of hydrogen peroxide at 240 nm [32]. Enzyme-specific activity was expressed in terms of U per milligram of protein.

The activity of glutathione peroxidase (GSH-Px-EC.1.11.1.6) [33] and glutathione reductase (GSSG-R-EC.1.6.4.2) [34] was determined by means of the spectrophotometric method at a wavelength of 340 nm, assessing the conversion of NADPH to NADP+ or monitoring the oxidation of NADPH. The activity of GSH-Px and GSSG-R was expressed in terms of mU per milligram of protein.

Thioredoxin reductase activity (TrxR-EC.1.8.1.9) was assessed colourimetrically at 412 nm using a commercial assay kit (Sigma–Aldrich, St. Louis, MO, USA) [35]. Enzyme activity was expressed in terms of U per milligram of protein. 

#### 2.2.5. Non-Enzymatic Antioxidant Levels

An enzyme-linked immunosorbent assay (ELISA) was used to determine the level of thioredoxin (Trx) [36] as described in the previous work. In short, keratinocytes and fibroblast suspension together with the anti-thioredoxin antibody (LOT No GR3209156-6, Abcam, Cambridge, MA, USA) were incubated overnight. Goat anti-rabbit/mouse antibody (LOT No 11246748, EnVision + Dual Link/HRP (horseradish peroxidase)) (Agilent Technologies, Santa Clara, CA, USA) was then added to the sample, incubated for 1 h in order to subsequently add chromogen (tetramethylbenzidine). The reaction was discontinued after sulphuric acid had been added, the absorbance having been measured at a wavelength of 450. The results were expressed in terms of µg per milligram of protein.

The level of reduced glutathione (GSH) in skin cells was determined by means of capillary electrophoresis (CE) [37] immediately after the sample preparation using an ultraviolet detector (200 nm). The level of the GSH was determined on the basis of a calibration curve (1–120 nmol/mL; r^2^ = 0.9984) and expressed in terms of nmol per milligram of protein.

#### 2.2.6. Protein Expression

Protein expression was measured by means of the enzyme-linked immunosorbent assay (ELISA) [38] as previously described. In short, a blocking solution (5% skim milk in carbonate-binding buffer) and skin cell suspension were incubated for 3 h at 4 °C. Then, depending on the protein being tested, an appropriate primary antibody was applied to it (each antibody was diluted 1:1000) against NFκB (p52, p65)(LOT No 3270702, 207056), TNFα (LOT No. 3792082), HO-1 (heme oxygenase-1, LOT No.SLBD2522V) (host: mouse) (Sigma–Aldrich, St. Louis, MO, USA), phospho-Nrf2 (nuclear factor erythroid 2-related factor 2, phosphorylated at Ser40, LOT No. WD3250083) (host: rabbit) (Santa Cruz Biotechnology, Heidelberg, Germany, and the samples were incubated overnight. Goat anti-rabbit/mouse antibody (EnVision + Dual Link/HRP (horseradish peroxidase)) was then added to the sample, and incubated for 1 h in order to subsequently add chromogen (tetramethylbenzidine) to it. The reaction was discontinued after sulphuric acid had been added, the absorbance having been measured at a wavelength of 450 (within 10 min). The obtained results were calculated according to standard curves for each protein (NFκB-p52; Lifespan Biosciences, Seattle, WA, USA, LLQ = 0.5 ng/mL; NFκB-p65; OriGene Technologies, Rockville, MD, USA, LLQ = 0.02 ng/mL; TNFα; Merck, Darmstadt, Germany, LLQ = 0.4 ng/mL; pNrf2; human Nrf2 (phospho S40), Belgium Gentaur BV, Kampenhout, Belgium, LLQ = 1.0 µg/mL; and HO-1; Enzo Life Sciences, Ann Arbor, MI, USA. LLQ = 0.6 ng/mL). 

#### 2.2.7. Lipid Peroxidation

Gas chromatography coupled with mass spectrometry (GC-MS/MS, Agilent Technologies, Santa Clara, CA, USA) was used to determine the level of 4-hydroxynonenal (4-HNE) [39] as clearly described earlier. In short, after the incubation of cell lysates with O-(2,3,4,5,6-pentafluorobenzyl)hydroxylamine hydrochloride, the sample was extracted with hexane and evaporated to dryness. Before injection onto the column, the samples were dissolved in N,O-bis(trimethylsilyl)trifluoroacetamide. The following ions were monitored during the analysis: m/z 333.0 and 181.0 for 4-HNE-PFB-TMS and m/z 307.0 for IS derivatives. 4-HNE levels were expressed in terms of nmol per milligram of protein.

#### 2.2.8. Protein modifications 

Oxidative protein modifications were assessed based on the level of 4-HNE-protein adducts (4-HNE-protein) [40] and changes in the level of carbonyl groups (CBO) [39]. 4-HNE protein adducts were determined by means of ELISA as described above. 4-HNE-His monoclonal antibody (mouse anti-4-HNE-His monoclonal antibody, 4-HNE clone 1g4) was used for that purpose. In order to determine the level of 4-HNE-protein adducts, a calibration curve ranging from 1 to 7 μmol/L, r^2^ = 0.9982 (LLQ = 1 μmol/L) was used. The level of 4-HNE-protein was expressed in terms of pg per milligram of protein.

The level of carbonyl groups (CBO) was determined spectrophotometrically (370 nm) [41]. Their level was determined on the basis of a calibration curve within the range from 0 to 2 mg/mL (r^2^ = 0.9996) and expressed in terms of nmol per mg of protein. 

#### 2.2.9. Immunofluorescence Staining and Confocal Microscopy

Cells were cultured on black wells in a 96-well plate at 0.01 × 10^6^ cells/well. After 24 h, the culture media were removed, the plate was washed with PBS, and 100 µL fresh medium containing the studied substances was added into the well. The cells were treated as described above. After 24 h, the culture media containing substances were removed and the cells were fixed with 3.7% formaldehyde solution at room temperature for 10 min. Later, permeabilisation with 0.1% Triton X-100 solution—a 10-min step—was performed. After the permeabilisation, the plate was washed twice with PBS and 3% FBS was used as a blocking agent at room temperature for 30 min. After the removal of 3% FBS, 50 µL of mouse monoclonal COL1A antibody (Santa Cruz Biotechnology, Heidelberg, Germany) diluted in 3% FBS (1:50 dilution) was added and the plate was incubated for one hour at room temperature. Then, at 50 µL per well, fluorescent (Alexa 488) anti-mouse secondary antibodies (Santa Cruz Biotechnology, Heidelberg, Germany) dissolved in 3% FBS (1:1000 dilution) were added and incubated for 1 h.

During this step, the plate was covered to protect it from light. When the secondary antibody solution was removed, the plate was washed 3 times with PBS and the wells were filled with 100 µL PBS containing 2 µg/mL Hoechst 33342 for the nuclei staining. The plate was visualised using the BD Pathway 855 Bioimaging system.

#### 2.2.10. Statistical Analysis

All the obtained data are expressed in terms of the mean ± SD (for *n* = 5) and analysed by means of the one-way ANOVA with Tukey’s post hoc test, used for multiple comparisons to determine the significant differences between the groups. A *p*-value < 0.05 was considered statistically significant.

## 3. Results

### 3.1. The Effect of CBG, CBD, and CBG+CBD on The Pro-Oxidative Parameters of Control and UVA-Irradiated Keratinocytes and Fibroblasts

**Figure 1:**
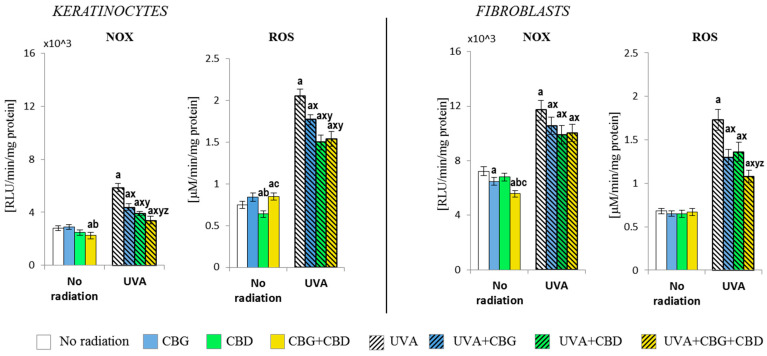
The effects of cannabigerol (CBG) (1 µM) or/and cannabidiol (CBD) (5 µM) on NADPH oxidase (NOX) and reactive oxygen species (ROS) generation in keratinocytes and fibroblasts in control groups and cells exposed to UVA radiation (keratinocytes-30 J/cm^2^ and fibroblasts-20 J/cm^2^). Mean values ± SD of five independent experiments are presented. The statistically significant differences in keratinocytes and fibroblasts are expressed as follows: a—indicates a comparison to the control group (control vs. all groups); *p* ≤ 0.05; b—indicates a comparison to the CBG group (only in no radiation part); *p* ≤ 0.05; c—indicates a comparison to the CBD group (only in no radiation part); *p* ≤ 0.05; x—indicates a comparison to the UVA radiation (only in UVA part); *p* ≤ 0.05; y—indicates a comparison to the UVA+CBG (only in UVA part); *p* ≤ 0.05; z—indicates a comparison to the UVA+CBD (only in UVA part); *p* ≤ 0.05.

The results have shown that CBG and CBD, used separately and in combination (CBG+CBD), differently influence the pro-oxidant abilities of both human keratinocytes and dermal fibroblasts (Figure 1). It was found that the NADPH oxidase activity was partially but significantly (*p* < 0.05) reduced by the combined CBD+CBG in control keratinocytes; meanwhile, in control fibroblasts, the reduction in NOX activity was caused by CBG. This was similar to keratinocytes regarding both phytocannabinoids if applied together. Despite the tendency to reduce the NOX activity, the ROS level was reduced only after the CBD had been applied to the keratinocyte medium; meanwhile, in the case of fibroblasts, none of the phytocannabinoids used either individually or in a two-component system influenced their ROS levels.

The exposure of both cell types to the UVA radiation resulted in an increase in NOX activity (over two-fold in keratinocytes and approximately 1.5-fold in fibroblasts). The addition of the phytocannabinoids into the culture medium after the UVA irradiation of cells resulted in an effective reduction in NOX activity in keratinocytes and a smaller but also significant reduction in the activity of that enzyme in fibroblasts, with the consequently limited production of ROS. CBD had the most effective effect on keratinocytes, while in the case of fibroblasts, both phytocannabinoids (CBG+CBD) had the strongest reducing effect. Hence, the observed changes in the NOX activity and ROS levels in the control group and in the UVA-irradiated keratinocytes, after the use of the phytocannabinoids, suggest a differentiated response due to the effect of the phytocannabinoids on different ROS-producing cellular systems.

### 3.2. The Effects of CBG, CBD, and CBG+CBD on the Antioxidant Efficiency of Control and UVA-Irradiated Keratinocytes and Fibroblasts

**Figure 2:**
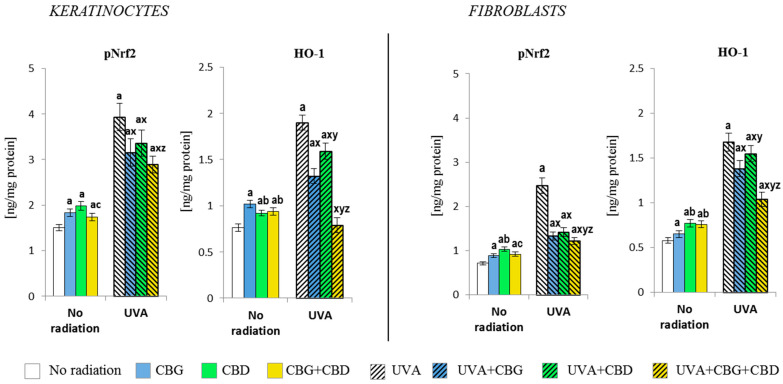
Effects of cannabigerol (CBG) (1 µM) or/and cannabidiol (CBD) (5 µM) on phorylated Nrf2 (p-Nrf2) and heme oxygenase-1 (HO-1) generation in keratinocytes and fibroblasts exposed to UVA radiation (30 J/cm^2^ and 20 J/cm^2^, respectively). The mean values ± SD of five independent experiments are presented. The statistically significant differences in keratinocytes and fibroblasts are expressed as follows: a—indicates a comparison to the control group; *p* ≤ 0.05; b—indicates a comparison to the CBG group; *p* ≤ 0.05; c—indicates a comparison to the CBD group; *p* ≤ 0.05; x—indicates a comparison to the UVA radiation; *p* ≤ 0.05; y—indicates a comparison to the UVA+CBG (only in UVA part); *p* ≤ 0.05; z—indicates a comparison to the UVA+CBD; *p* ≤ 0.05.

**Figure 3:**
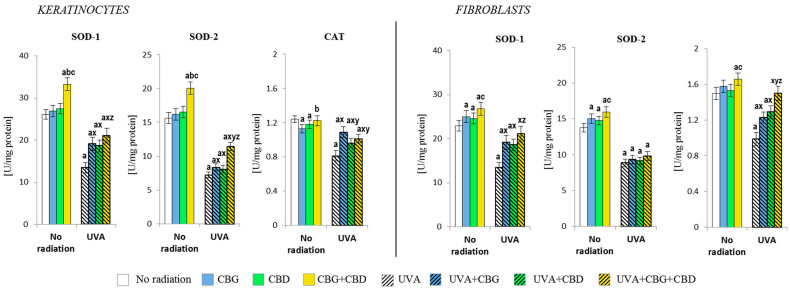
Effects of cannabigerol (CBG) (1 µM) or/and cannabidiol (CBD) (5 µM) on superoxide dismutase (SOD-1 and SOD-2) and catalase (CAT) activity in keratinocytes and fibroblasts exposed to UVA radiation (30 J/cm^2^ and 20 J/cm^2^), respectively. The mean values ± SD of five independent experiments are presented. The statistically significant differences in keratinocytes and fibroblasts are expressed as follows: a—indicates a comparison to the control group; *p* ≤ 0.05; b—indicates a comparison to the CBG group; *p* ≤ 0.05; c—indicates a comparison to the CBD group; *p* ≤ 0.05; x—indicates a comparison to the UVA radiation; *p* ≤ 0.05; y—indicates a comparison to the UVA+CBG (only in UVA part); *p* ≤ 0.05; z—indicates a comparison to the UVA+CBD; *p* ≤ 0.05.

**Figure 4:**
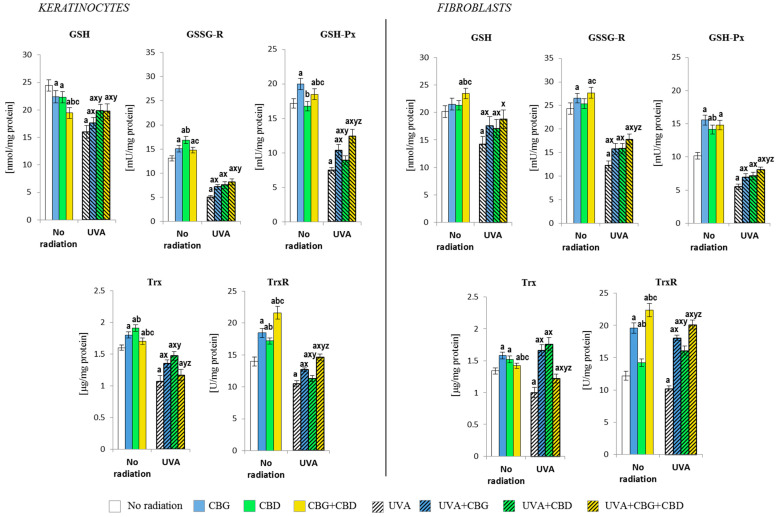
Effects of cannabigerol (CBG) (1 µM) or/and cannabidiol (CBD) (5 µM) on a glutathione (GSH, GSSG-R, GSH-Px) and thioredoxin (Trx, TrxR)-dependent system in keratinocytes and fibroblasts exposed to UVA radiation (30 J/cm^2^ and 20 J/cm^2^, respectively). The mean values ± SD of five independent experiments are presented. The statistically significant differences in keratinocytes and fibroblasts are expressed as follows: a—indicates a comparison to the control group; *p* ≤ 0.05; b—indicates a comparison to the CBG group; *p* ≤ 0.05; c—indicates a comparison to the CBD group; *p* ≤ 0.05; x—indicates a comparison to the UVA radiation; *p* ≤ 0.05; y—indicates a comparison to the UVA+CBG (only in UVA part); *p* ≤ 0.05; z—indicates a comparison to the UVA+CBD; *p* ≤ 0.05.

Both individually used CBG and CBD and their combination (CBG+CBD) influenced the antioxidant capacities of keratinocytes and fibroblasts, which was already visible at the level of the expression of the Nrf2 transcription factor responsible for the bio-synthesis of antioxidants and at the level/activity of antioxidant proteins and glutathione (Figure 2, Figure 3 and Figure 4). The application of the phytocannabinoids to the medium of both types of the control, non-irradiated, cells resulted in an increase in the level of pNrf2 (most effectively after the use of CBD) and the level of the basic product of its transcriptional activity, i.e., heme oxygenase-1 (HO-1). The greatest increase was observed in keratinocytes after the use of CBG and in fibroblasts after using CBD or the combination of both phytocannabinoids (Figure 2). The UVA irradiation also caused a significant, higher than two-fold, increase in the levels of pNrf2 and HO-1 in both keratinocytes and fibroblasts. However, the phytocannabinoids applied to the medium of the cells previously exposed to the UVA radiation showed a regenerative effect by significantly reducing (especially in fibroblasts) the expression of pNrf2, which was especially pronounced when both phytocannabinoids were used concurrently. The same direction of changes was observed for the HO-1 levels, while the CBG+CBD combined again worked most effectively, reducing the HO-1 level in keratinocytes even to the values observed in the controls.

The changes observed at the level of the Nrf2 transcription factor caused by the phytocannabinoids also promoted the changes in the level/activity of the antioxidants that had been applied (Figure 3 and Figure 4). It has been found that especially the CBG+CBD system, and to a lesser extent, the respective phytocannabinoids, significantly increased the activity of both isoforms of superoxide dismutase, i.e., cytosolic (SOD-1) and mitochondrial (SOD-2), in keratinocytes, and, to a lesser extent, in fibroblasts. Moreover, when both phytocannabinoids were concurrently applied, they also increased the catalase activity in fibroblasts (Figure 3). However, the UVA irradiation of both cell types resulted in a significant reduction in the activity of the abovementioned enzymes in keratinocytes and fibroblasts. The addition of the phytocannabinoids, especially CBG+CBD, to the medium of irradiated cells had beneficial effects on the skin cells, favouring the reversal of the changes induced by the UVA radiation. In the case of SOD-1 and CAT of fibroblasts, the CBG+CBD combination led to reversibility in the activity of both enzymes to the respective control values.

The phytocannabinoids also caused changes in the level/activity of the components of the GSH- and Trx-dependent antioxidant systems (Figure 4). The combination of the two phytocannabinoids (CBG+CBD) in the medium of non-irradiated keratinocytes reduced the level of GSH, increasing the activity of enzymes relevant to the function of this antioxidant tripeptide (GSH-Px and GSSGR). Furthermore, CBG has been found to be the most effective on GSH-Px, while CBD was more effective on the GSSG-R activity. However, the phytocannabinoids, having been added to the fibroblast culture medium, triggered a different metabolic response. Namely, the level of GSH and the activity of GSSGR and TrxR were most strongly increased by the combination of both phytocannabinoids, while the greatest increase in the levels of Trx and GSHPx was caused by CBG applied alone.

Both in keratinocytes and in fibroblasts exposed to the UVA, a reduction in the level/activity of all the parameters of GSH- and Trx-dependent antioxidant systems were observed, while the addition of the phytocannabinoids reduced those irradiation effects. In the case of the GSH-dependent system, the phytocannabinoids—when concurrently applied—worked most effectively, both in keratinocytes and fibroblasts. However, in the case of the Trx-dependent system, the most effective was CBD applied alone, while the activity of TrxR was most strongly enhanced by the combined CBG+CBD treatment.

**Figure 5:**
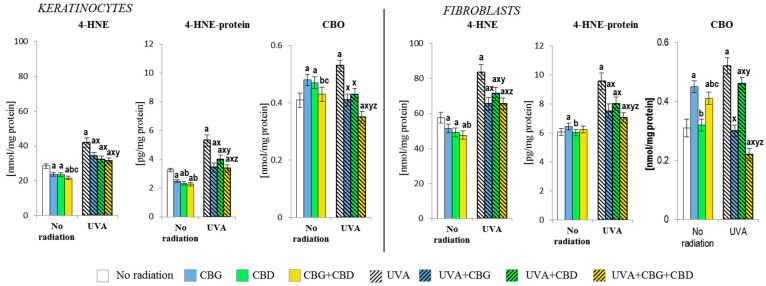
Effects of cannabigerol (CBG) (1 µM) or/and cannabidiol (CBD) (5 µM) on 4-hydroxynonenal (4-HNE) and 4-hydroxynonenal-protein (4-HNE-protein) generation, and the level of carbonyl group (CBO) in keratinocytes and fibroblasts exposed to UVA radiation (30 J/cm^2^ and 20 J/cm^2^, respectively). The mean values ± SD of five independent experiments are presented. The statistically significant differences in keratinocytes and fibroblasts are expressed as follows: a—indicates a comparison to the control group; *p* ≤ 0.05; b—indicates a comparison to the CBG group; *p* ≤ 0.05; c—indicates a comparison to the CBD group; *p* ≤ 0.05; x—indicates a comparison to the UVA radiation; *p* ≤ 0.05; y—indicates a comparison to the UVA+CBG (only in UVA part); *p* ≤ 0.05; z—indicates a comparison to the UVA+CBD; *p* ≤ 0.05.

Shifting the redox balance in the control keratinocytes and fibroblasts towards the reduction reaction as a result of the action of CBG, CBD, and, especially, CBG+CBD decreased the onset of the cellular lipid peroxidation assessed by means of the level of 4-hydroxynonenal (4-HNE, Figure 5). The phytocannabinoids reduced the protein modification caused by 4-HNE in keratinocytes; however, this effect was not observed in fibroblasts, in which CBG even slightly increased the level of the 4-HNE-protein adducts. The levels of the protein carbonyl groups were also increased by CBG, even in both types of cells; meanwhile, in keratinocytes, the effects of CBD were also similar. However, the use of the combined CBG+CBD did not increase the values of protein carbonyls in fibroblasts.

The shift of the redox balance towards oxidation through UVA irradiation enhanced the reactions of ROS with lipids and proteins. This resulted in increased lipid peroxidation, which was reflected in the increased levels of 4-HNE and, subsequently, increased the levels of 4-HNE-protein adducts in both keratinocytes and fibroblasts to a relatively similar extent. However, the addition of the phytocannabinoids into the culture medium after the irradiation of cells with the UVA resulted in a reduction in both the level of 4-HNE itself and 4-HNE-protein adducts, with the CBG+CBD combination acting most effectively. Additionally, the use of both compounds in combination resulted in the greatest reduction in the level of carbonyl groups in proteins, especially in fibroblasts, where a reduction in the level of carbonyl groups was found for the UVA-treated cells to be even below the control values.

**Figure 6:**
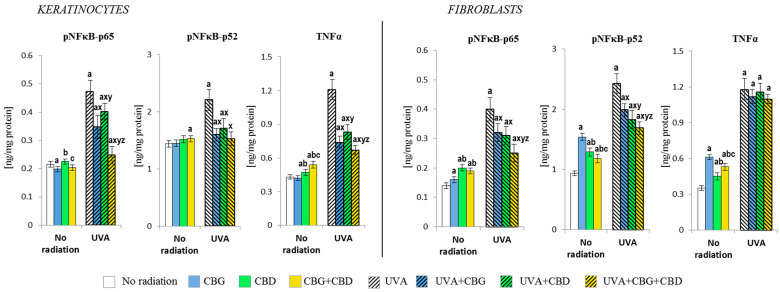
Effects of cannabigerol (CBG) (1 µM) or/and cannabidiol (CBD) (5 µM) on nuclear factor kappa-light-chain-enhancer of activated B cells (NFκB-p65 and NFκB-p52) and tumour necrosis factor-alpha (TNFα) generation in keratinocytes and fibroblasts exposed to UVA radiation (30 J/cm^2^ and 20 J/cm^2^, respectively). Mean values ± SD of five independent experiments are presented. The statistically significant differences in keratinocytes and fibroblasts are expressed as follows: a—indicates a comparison to the control group; *p* ≤ 0.05; b—indicates a comparison to the CBG group; *p* ≤ 0.05; c—indicates a comparison to the CBD group; *p* ≤ 0.05; x—indicates a comparison to the UVA radiation; *p* ≤ 0.05; y—indicates a comparison to the UVA+CBG (only in UVA part); *p* ≤ 0.05; z—indicates a comparison to the UVA+CBD; *p* ≤ 0.05.

The phytocannabinoids further modified the levels of the Nrf2 transcription factor, thus influencing the response of the NFκB transcription factor (studied as its subunit) and, consequently, the proinflammatory cellular response (Figure 6). In the case of control keratinocytes, the reduced level of the p65 subunit was visible after the CBG treatment, while, after the CBD treatment, the p65 level increased. In fibroblasts, both phytocannabinoids used alone or in combination increased the p65 levels. However, in the case of keratinocytes p52, only the phytocannabinoids concurrently applied (CBG+CBD) increased its level; meanwhile, in fibroblasts, all phytocannabinoids-based treatments increased the level of p65, with CBG being the most effective. As a consequence, the changes in the level of the product efficiency of the transcription factor—the cytokine TNFα—were also observed in the control cells. Its levels increased in keratinocytes after the use of CBD and CBG+CBD concurrently, while, in fibroblasts, the level of TNFα increased even more after the use of CBG alone and slightly less after CBG+CBD combined.

The UVA irradiation significantly induced the expression of the subunits of the proinflammatory transcription factor NFκB (p65 and p52) and the amounts of TNFα, while such effects of the UVA were partially counteracted by the phytocannabinoids. In keratinocytes, the changes of both subunits of the NFκB were similar, while the p65 subunit was the most strongly inhibited by the combined phytocannabinoids, whereas CBD alone was the least effective. Similar changes were also observed in fibroblasts, with CBG being the least effective. The TNFα levels in irradiated keratinocytes were effectively reduced by the phytocannabinoids, while in the case of fibroblasts, the use of the phytocannabinoids did not significantly change the TNFα level.

**Figure 7:**
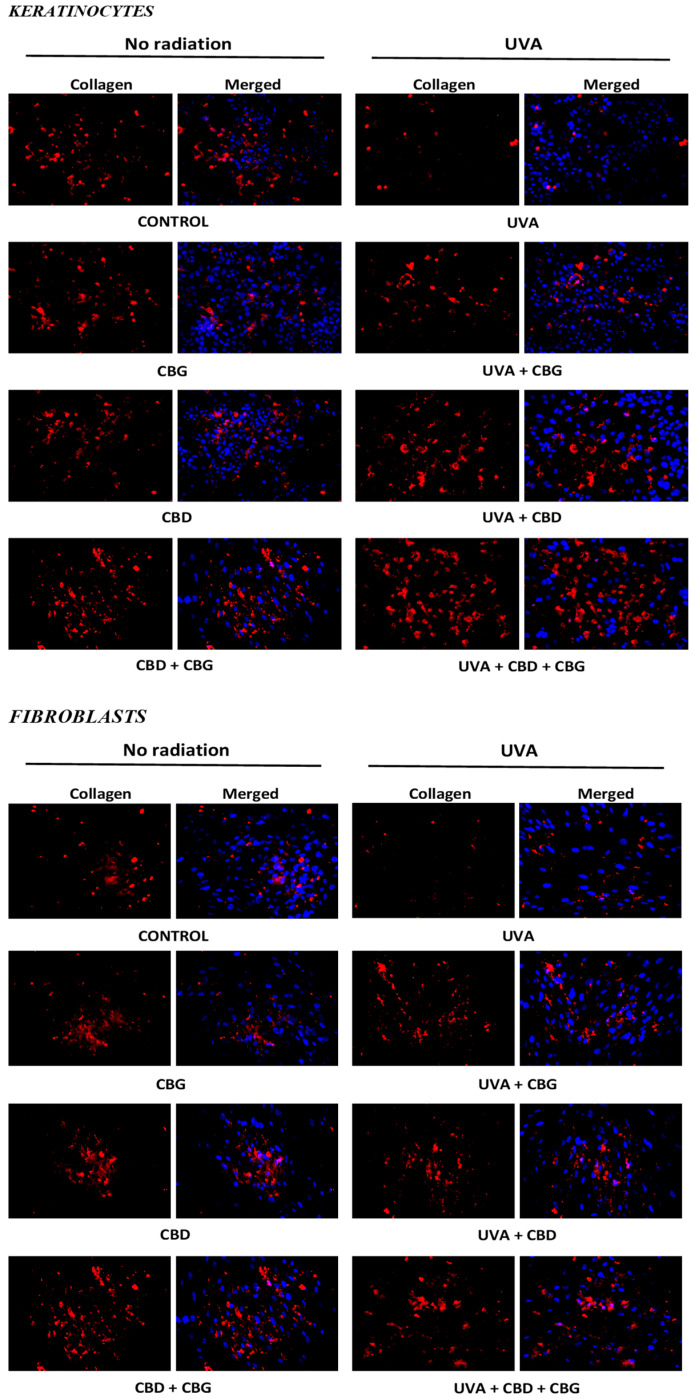
Effects of cannabigerol (CBG) (1 µM) or/and cannabidiol (CBD) (5 µM) on collagen expression in keratinocytes (*n* = 3) and fibroblasts (*n* = 3) exposed to UVA radiation (30 J/cm^2^ and 20 nJ/cm^2^, respectively).

Regardless of the change in protein modification caused by 4-HNE, the individually used CBD did not affect the expression of type I collagen in the tested cells, while CBG slightly reduced its expression. However, when CBG and CBD were concurrently applied (CBG+CBD), they increased the expression of that protein, which may indicate the synergistic effect of both compounds (Figure 7). Since the UVA irradiation caused a significant reduction in the expression of type I collagen in both keratinocytes and fibroblasts, the use of the phytocannabinoids, especially their combination (CBG+CBD), had a regenerative effect, reducing the damage at the level of protein biosynthesis caused by the UVA radiation.

## 4. Discussion

Although the related literature suggests the therapeutic potential of phytocannabinoids, their regulatory effects on skin cells have so far been focused mainly on CBD. Both in vitro and in vivo studies have shown that CBD has protective and regenerative effects on skin cells exposed to both physical and chemical factors [4,42,43], and its antiproliferative and anti-inflammatory effects have already been confirmed in clinical trials conducted on patients with psoriasis or atopic dermatitis [44]. However, the experimental data regarding the metabolic effects of other phytocannabinoids, mainly CBG, show, among other things, anti-inflammatory effects in various metabolic diseases [45,46], and, thus, prevent the proinflammatory effects of exogenous physicochemical factors on skin cells, suggesting the possible use of this cannabinoid too [22,47]. Moreover, CBG has also been shown to have antioxidant properties [15]. Therefore, it is suggested that CBG, by modifying metabolic mechanisms other than CBD, may protect the phospholipid structures of cell membranes more effectively than CBD [25], which is also partly in agreement with the results of this study.

**Figure 8:**
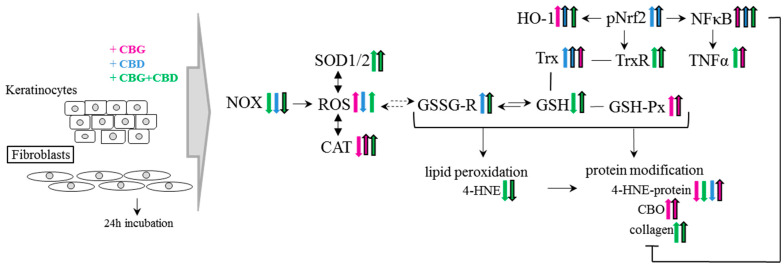
The effect of phytocannabinoids on keratinocytes and fibroblasts under standard conditions. Abbreviation: 4-HNE, 4-hydroxynonenal, 4-HNE-protein, 4-HNE-protein adducts; CAT, catalase; CBD, cannabidiol; CBG, cannabigerol; CBO, carbonyl groups; GSH, glutathione; GSH-Px, glutathione peroxidase; GSSG-R, glutathione reductase; HO-1, heme oxygenase-1; NF-κB, nuclear factor kappa-light-chain-enhancer; NOX, NADPH oxidase; Trx, hioredoxin; pNrf2, nuclear factor erythroid 2-related factor 2 phosphorylated form; ROS, reacctive oxygene species; SOD-1, cytosolic superoxide dismutase; SOD-2, mitochondrial superoxide dismutase; TNFα, tumor necrosis factor α; TrxR, thioredoxin reductase; UV, ultraviolet radiation.

Namely, the results of our study have revealed the regulatory effect of the phytocannabinoids, and especially the combined use of the two phytocannabinoids (CBG+CBD), on the redox balance even in the control keratinocytes and skin fibroblasts not exposed to UVA irradiation (Figure 8). Those effects involve a reduction in NOX activity (the basic cellular enzyme responsible for the generation of superoxide anion radical) by CBD in keratinocytes. CBG had a similar effect, but in fibroblasts and, consequently, in both types of skin cells, the use of the two phytocannabinoids (CBG+CBD) has been found to be the most effective. Therefore, it seems that phytocannabinoids can effectively protect the cells of the epidermis and dermis too. The effectiveness of the used phytocannabinoids may arise from their lipophilic nature and, thus, their possible influence on the NOX present in the cellular membrane [48]. On the other hand, the increase in the ROS levels combined with the reduced NOX activity in keratinocytes after the use of CBG and CBG+CBD concurrently applied with the lack of reduction in ROS with the reduced NOX activity in fibroblasts may also indicate that the phytocannabinoids, especially CBG, may modify the other metabolic pathways leading to ROS production, including the mitochondrial chain reactions. This is in agreement with the previous findings on CBD and other phytocannabinoids that stimulated ROS generation through the mitochondrial system [49].

Moreover, the increased ROS production in keratinocytes may also be the consequence of the increased activation of G-protein-coupled receptors (mainly CB1 and CB2) by the phytocannabinoids that are involved in, among other processes, the regulation of redox homeostasis [50]. The increased ROS production may be the result of the activation of the CB1 membrane receptor in keratinocytes, the increased expression of which under the influence of phytocannabinoids has been previously observed [20,51]. In several cell types, the CB1 receptor is expressed both on the cell membrane and on the outer membrane of mitochondria, modifying the mitochondrial functions of human epidermal cells [52], which may lead to the overproduction of ROS. In response to such effects as the phytocannabinoids modelling the level of ROS in skin cells, cellular basic antioxidant enzymes responsible for the ROS metabolism may also be involved, in particular superoxide dismutase isoenzymes occurring both in the cytosol and mitochondria (SOD-1 and SOD-2) of keratinocytes and fibroblasts, the activity of which increases especially after using the CBG+CBD system. A similar response to the combined effect of CBG and CBD applies to the fibroblast catalase. Previous literature data indicated the same direction of modification of the activity of basic antioxidant enzymes after the use of CBD [53].

Both these results and previous literature data regarding CBD [53] indicate that this phytocannabinoid modulates the redox balance and, consequently, has a cytoprotective effect mainly by modifying antioxidant signalling [54]. This includes the changes in the antioxidant capabilities of cellular peptides/proteins at the level of their transcription regulated by Nrf2 [54]. The results of this study confirm the existing data on the effect of CBD [53] but also show that CBG, acting individually, but especially in combination with CBD, increases both the expression and transcriptional efficiency of Nrf2 as assessed by the level of HO-1, which, like the products, its degradation has an antioxidant and anti-inflammatory effect in both types of skin cells used [55]. It is also known that HO-1 over-expression promotes a reduction in ROS and inflammatory mediators in keratinocytes [56], which makes both heme oxygenase itself and its inducers potential therapeutic targets in skin diseases [57]. The increased Nrf2 expression should promote a reduction in ROS levels in cells [58]. However, the observed increase in ROS suggests that the phytocannabinoids enhance the generation of mitochondrial ROS, thereby inducing the feedback action of the antioxidant system.

The obtained results also indicate that the use of phytocannabinoids, especially the CBG+CBD combination, may modify the protection of membrane phospholipids. This is manifested by an increase in the level/activity of the antioxidant components of the Try-dependent system and, to a lesser extent, the GSH-dependent system [4]. The Trx-dependent system is particularly important in the detoxification of harmful metabolites, such as lipid peroxides, as well as in the regulation of the gene expression and modulation of cellular signalling pathways [59]. It has previously been shown that CBD supports the Trx-dependent system, leading to the increased expression/activity of its components [4]. The results of the current study are consistent with those findings and indicate that thioredoxin reductase, which is highly expressed in human keratinocytes, is particularly susceptible to CBG, suggesting that the Trx system may be the primary CBG mechanism protecting skin cells, especially lipid structures, against the destructive effects of ROS [60]. It has been suggested that the Trx system influences cell survival by preventing inflammation, especially in keratinocytes [61], which is also consistent with our results. The protective effects of the phytocannabinoids used in this study included at least a partial reduction in lipid peroxidation, observed particularly in keratinocytes, as indicated by the reduced levels of reactive 4-HNE aldehyde [4,62]. This proves the protective effect of the phytocannabinoids used (especially for the CBG+CBD system). As a consequence, a partially protective effect of that system on cellular proteins is also observed, protecting them against modifications caused by 4-HNE [43].

The phytocannabinoids, by modifying the Nrf2 microenvironment, promote the interaction of the inhibitor of that transcription factor—Kelch-like ECH-related protein 1 (Keap1)—with a transcription factor that regulates inflammatory processes, such as NFκB [53,54]. It has previously been shown that CBD tends to increase the expression of TNFα in keratinocytes (HaCaT), thus promoting an increase in NFκB levels [62], which is confirmed by these results, while showing a stronger response for fibroblasts than keratinocytes. It has previously been shown in non-cellular contexts that CBG, by reducing the phosphorylation of Iκβ-α, an NFκB inhibitor [63,64], also increases the transcriptional activity responsible for the biosynthesis of proinflammatory cytokines, including TNFα and IL-1β [23]. However, the obtained results may suggest that the modulation of the NFκB signalling pathway, mediated by the phytocannabinoids (especially CBG), depends on the cell type.

**Figure 9:**
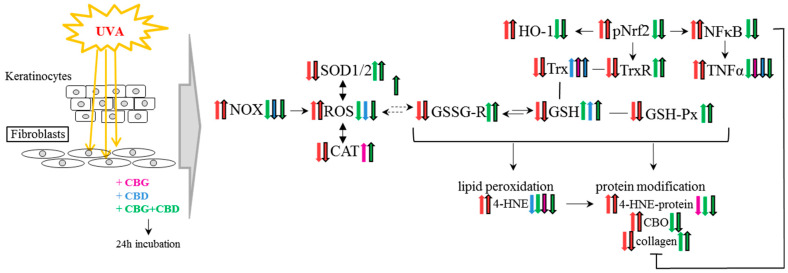
The effect of phytocannabinoids on keratinocytes and fibroblasts used after UVA cell irradiation. Abbreviation: 4-HNE, 4-hydroxynonenal, 4-HNE-protein, 4-HNE-protein adducts; CAT, catalase; CBD, cannabidiol; CBG, cannabigerol; CBO, carbonyl groups; GSH, glutathione; GSH-Px, glutathione peroxidase; GSSG-R, glutathione reductase; HO-1, heme oxygenase-1; NF-κB, nuclear factor kappa-light-chain-enhancer; NOX, NADPH oxidase; Trx, hioredoxin; pNrf2, nuclear factor erythroid 2-related factor 2 phosphorylated form; ROS, reacctive oxygene species; SOD-1, cytosolic superoxide dismutase; SOD-2, mitochondrial superoxide dismutase; TNFα, tumor necrosis factor α; TrxR, thioredoxin reductase; UV, ultraviolet radiation.

The obtained results have proven that exposure to the UVA rays significantly changes the response of skin cells to the phytocannabinoids (Figure 9). The UVA modulates the cellular metabolism of both keratinocytes and fibroblasts [65], including, among other things, the increase in the production of ROS, which favours the modification of the redox balance and the intensification of inflammatory processes, as previously demonstrated [66,67]. This leads to an increased risk of oxidative photodamage, as demonstrated in the previous studies using skin cell cultures in both 2D and 3D cultures [4,43,62]. These results show that NOX activity is significantly lower in keratinocytes exposed to UVA radiation than in fibroblasts, while the ROS level in keratinocytes is higher than in fibroblasts. This paradox points to the mitochondrial electron transport chain as a source of ROS and oxidative stress in keratinocytes, as previously demonstrated by assessing the release of cytochrome c into the cytosol of keratinocytes exposed to UV radiation [68]. Both literature data [4,22,69] and the results of this research indicate that CBG and CBD, especially when concurrently used, may significantly reduce NOX activity and the level of ROS increased by UVA radiation. Previous studies have also shown that CBG reduces the ROS levels in rat fibroblasts and astrocytes, elevated by the pro-oxidant effects of hydrogen peroxide [70], and also has a protective effect on keratinocytes and fibroblasts exposed to the UVA/B radiation [69]. Both CBG and CBG+CBD have been found to influence the redox balance by inhibiting the activity of inducible nitric oxide synthase (iNOS), which is one of the main pro-oxidant factors activated by proinflammatory factors (e.g., LPS) [63].

It should also be taken into account that the stimulation of cellular metabolism, including oxidative systems in cells, by the UVA, lasts longer than the direct effects of the exposure itself. Hence, the phytocannabinoids applied after the UVA rays, like other phenolic compounds, may be oxidised to form radical forms or may undergo oxidative degradation [69]. Those processes could potentially reduce the regenerative effects of the phytocannabinoids or even increase the overall oxidative stress, causing an “entourage effect”. Taking into consideration that the redox stability of CBG is lower than that of CBD [69], it may be the reason for the increased level of ROS in keratinocytes after the use of CBG+CBD and the lack of changes after the application of the phytocannabinoids to the fibroblast medium. Previously, it was also found that the biological activity of the oxidised form of CBD varied after oxidation [71]; therefore, the oxidised CBD inhibited some enzymes, including topoisomerase II α and β, which was not observed for the native CBD alone [72]. It seems possible that similar processes may also influence NOX activity in fibroblasts. On the other hand, the lower stability of CBG in oxidising conditions may also result in the formation of a quinone derivative of CBG, the biological activity of which may be higher than that of CBD itself [73]. It has also been shown that the modified structures of the phytocannabinoids influence their binding affinity with CB1, CB2, and PPARγ receptors (peroxisome proliferator-activated receptor γ) [74], while the activation of those receptors results in, among other things, the modification of the level of the generated ROS and/or the activity of antioxidant enzymes involved in the regulation of the cellular redox homeostasis [50]. Moreover, the CB1 receptor is expressed both on the cell membrane and on the outer mitochondrial membrane of human epidermal cells under physiological conditions [52], which, as a consequence of the cell irradiation with the UVA rays, may lead to changes in ROS generation.

The UVA also leads to the modification of the antioxidant response of cells by changing the expression of the Nrf2 transcription factor. Moreover, previous studies have also shown that CBD reduces the oxidative stress and the UVA-induced proinflammatory signalling in keratinocytes and fibroblasts, both by reducing the ROS production, changes in the levels of the transcription factor Nrf2, and by modifying the activities of cellular peptide-protein antioxidant [4,43]. The results obtained in this study have shown that CBG, especially when applied concurrently with CBD, normalises the level of the transcription factor Nrf2, which is responsible for the biosynthesis of cytoprotective proteins [54,58] and the level of its transcriptional efficiency indicator, HO-1, which is significantly increased by the UVA, and this is clearly confirmed in the related literature [75]. Moreover, this is probably caused by the increased level of Nrf2 inhibitors, including cytosolic (Keap1) and nuclear (BTB and CNC 1 homology, Bach1), which was already observed in earlier studies after the application of CBD to the medium of keratinocytes previously exposed to the UV radiation [62]. Moreover, CBD and CBG, by decreasing the UV-induced 4-HNE level, reduce the possibility of 4-HNE–Keap1 binding, and, therefore, block the release of the Nrf2 molecule from the Nrf2–Keap1 complex. The application of phytocannabinoids after cell irradiation with UVA rays was accompanied by the increased activity of the main enzymatic antioxidants, including cytosolic (SOD-1; CAT) and mitochondrial SOD-2, which decreased as a result of the UVA irradiation. So far, the increase in SOD and CAT in skin cells has been observed only after the use of CBD [4,62], while the results of our research indicate the effectiveness of CBG and the synergism of the action of both phytocannabinoids, especially the increase in the activity of SOD-2 in keratinocytes and CAT in fibroblasts, which promotes a reduction in the level of ROS. Although CBD has already been thoroughly studied in human skin cells exposed to UV radiation [4,76], so far, only an increase in SOD-1/2 and CAT activity has been found in cells other than skin cells [20]. Therefore, our results present new arguments in favour of the possibility of using CBG, which has, so far, been proven to reduce the severity of neurological diseases associated with oxidative stress, such as Parkinson’s disease, amyotrophic lateral sclerosis, or microgliosis [49,73].

These results also show that CBG may restore the effectiveness of the GSH-dependent system of skin cells exposed to UVA radiation. Since the related literature indicates a high affinity of CBD for cysteines 288/151 of glutathione peroxidase [62], and taking into account the structural similarity of CBD and GBG, it is plausible that CBG also works effectively through interaction with that cysteine moiety or other structural elements of that enzyme, which is important for its activities. CBD also works effectively at the level of the antioxidant thioredoxin system by maintaining thioredoxin reductase in a reduced and metabolically effective form, which has already been demonstrated in the related literature [53]. This may suggest the existence of the synergistic mechanisms of action of respective antioxidants acting in the area of lipid protection within the GSH and Trx-dependent systems. Although, so far, there has been no literature on the effect of CBG on the antioxidant systems of human skin cells, it is known that CBD effectively supports the thioredoxin system of keratinocytes in vitro [62] and in the skin cells of rats exposed to the UVA/B radiation and treated with CBD in vivo [53], as well as keratinocytes isolated from patients with psoriasis (ex vivo) [4]. 

It can, therefore, be concluded that the tested phytocannabinoids complement one another in terms of their regenerative effects on both systems responsible for the protection of membrane phospholipids, which, especially in the case of the CBG+CBD system, results in a significant reduction in lipid peroxidation assessed by the level of 4-HNE and, consequently, also in a reduced level of 4-HNE-protein adducts. It is known that reducing the level of 4-HNE-protein adducts also significantly prevents the activation of proinflammatory factors and regulates intracellular signalling [77]. The observed decrease in the level of 4-HNE-protein adducts may affect the cytosolic inhibitor of the Nrf2 transcription factor, namely, the Keap1, as has been previously shown using CBD [54], and may promote not only the activation of Nrf2 but also a reduction in NFκB-dependent inflammation or p53-dependent apoptosis [78,79]. The analysis of the expression and biological effectiveness of the NFκB transcription factor (assessed by TNFa level) in this study has confirmed such antioxidant and anti-inflammatory activity principles of the phytocannabinoids.

The use of the CBG+CBD system, especially to treat keratinocytes previously exposed to the UVA, reduced the levels of NFκB subunits and its transcriptional product, the cytokine TNFα, which also plays a key role in skin photoaging [22,80]. Since NFκB also plays a significant role in the development of skin diseases such as cancer and psoriasis, while its expression is strongly induced by TNFα [81,82], reducing its expression by combining CBG+CBD might be beneficial to prevent those skin diseases [54]. The regenerative effect, especially of the combined CBG+CBD, in relation to the UVA-caused oxidative damage of proteins, was evidenced by the prominent reduction in carbonyl groups, even to a level lower than in the control cells not treated by the UVA. This is extremely important because the increase in the level of carbonyl groups is usually associated with a decrease in the biological effectiveness of proteins, including their antioxidant activity, which was observed in the case of GSH-Px and CAT in keratinocytes and fibroblasts.

Regardless of the assessment of the impact of the tested phytocannabinoids on proteins of the redox system and those involved in the development of inflammation, the response of collagen was also checked, which was an example of a protein on which the phytocannabinoids had both protective and regenerative effects. It has been found that, under the control conditions, CBG slightly increases collagen expression in both fibroblasts and keratinocytes, which may be related to the effect of CBG on collagen-degrading enzymes, including the stimulation of metalloproteinases [83]. However, the oxidative stress and proinflammatory conditions induced by the UVA radiation clearly inhibit collagen biosynthesis through various mechanisms [84]. The use of the phytocannabinoids, especially the combination of CBG+CBD, reduced the negative effect of UVA rays on collagen in skin cells, which may have been the result of inhibiting oxidative stress and proinflammatory factors inhibiting the biosynthesis of this protein. The related literature indicates that the synthetically obtained CBG has a much stronger effect than CBD, increasing the level of collagen (I, III, and IV) and elastin (in a 3D model of human skin), and, thus, among other things, improving skin hydration, strengthening its barrier properties, and slowing down ageing and the occurrence of skin diseases [85].

## 5. Limitations

The research results and their analysis are subject to certain limitations. One of them is the use of classic cell culture models, which, on the one hand, perfectly reflects the nature of the changes occurring in the metabolism of one type of cells under the standardised conditions of the prepared experiment. However, the results obtained in this model will not always correspond to the actual reactions of the cells that, in natural conditions, occur in multilayers as well as in the neighbourhood of other cell types with which they also interact. In this case, the multilayer structure of the skin is also permeable to UVA radiation to varied degrees, which also limits the application of the results from the single-layer culture to the complex structure of the skin. Moreover, under natural conditions, the availability of the protective substance for various layers of the skin is not as uniform as in the case of the culture medium surrounding cells in in vitro cultures.

The results obtained in this study, although they contribute to expanding the possibilities of skin protection by understanding the mechanisms of the action of the compounds used, provide the answer within two cell lines belonging to two layers of the skin at the same time, the dermis and the epidermis; however, they ignore their metabolic interactions with melanocytes or immune cells that the tested phytocannabinoids may also affect.

Another limitation of the presented experiment is the targeted and point-based approach to the analyses. A wider scope of research would allow for a more accurate view of the effects of the tested compound, but the scope of the research to be carried out as part of this project covered only selected aspects.

## 6. Conclusions

The results presented in this manuscript indicate that the concurrent use of the two phytocannabinoids (CBG and CBD), acting as both a protective and regenerative system, may have a beneficial effect on the redox balance in human keratinocytes and skin fibroblasts, even if they were applied after UVA irradiation. The tested phytocannabinoids also counteract proinflammatory reactions, which, consequently, contribute to the development of various pathological conditions. The obtained results suggest the combined use of CBG and CBD as a potential preventive and regenerative method for skin cells, especially those damaged by UV radiation, which may be used for the purpose of both prevention and therapy.

Additionally, it should be emphasised that the effect of CBG on skin cells is multidirectional, both in relation to proteins and lipids, which would be worth taking into account in the future when planning experiments with a broader methodological aspect, both in vitro and in vivo.

## Data Availability

All data generated or analysed during this study are included in this article.
